# Skin autofluorescence, reflecting accumulation of advanced glycation end products, and the risk of dementia in a population-based cohort

**DOI:** 10.1038/s41598-024-51703-6

**Published:** 2024-01-13

**Authors:** Sanne S. Mooldijk, Tianqi Lu, Komal Waqas, Jinluan Chen, Meike W. Vernooij, M. Kamran Ikram, M. Carola Zillikens, M. Arfan Ikram

**Affiliations:** 1 Department of Epidemiology, Erasmus University Medical Center, PO Box 2040, 3000 CA Rotterdam, The Netherlands; 2 Department of Internal Medicine, Erasmus University Medical Center Rotterdam, Rotterdam, The Netherlands; 3 Department of Radiology and Nuclear Medicine, Erasmus MC University Medical Center, Rotterdam, The Netherlands; 4 Department of Neurology, Erasmus University Medical Center Rotterdam, Rotterdam, The Netherlands

**Keywords:** Dementia, Diabetes

## Abstract

Conditions such as hyperglycemia and oxidative stress lead to the formation of advanced glycation end products (AGEs), which are harmful compounds that have been implicated in dementia. Within the Rotterdam Study, we measured skin AGEs as skin autofluorescence, reflecting long-term accumulation of AGEs, and determined their association with the risk of dementia and with brain magnetic resonance imaging (MRI) measures. Skin autofluorescence was measured between 2013 and 2016 in 2922 participants without dementia. Of these, 1504 also underwent brain MRI, on which measures of brain atrophy and cerebral small vessel disease were assessed. All participants were followed for the incidence of dementia until 2020. Of 2922 participants (mean age 72.6 years, 57% women), 123 developed dementia. Higher skin autofluorescence (per standard deviation) was associated with an increased risk of dementia (hazard ratio 1.21 [95% confidence interval 1.01–1.46]) and Alzheimer’s disease (1.19 [0.97–1.47]), independently of age and other studied potential confounders. Stronger effects were seen in apolipoprotein E (*APOE)* ε4 carriers (1.34 [0.98–1.82]) and in participants with diabetes (1.35 [0.94–1.94]). Participants with higher skin autofluorescence levels also had smaller total brain volumes and smaller hippocampus volumes on MRI, and they had more often lacunes. These results suggest that AGEs may be involved in dementia pathophysiology.

## Introduction

Advanced glycation end products (AGEs) are the final products of proteins or lipids that become glycated and oxidized^1,2^. They are primarily formed in environments of hyperglycemia and oxidative stress but also accumulate with normal aging. AGE levels are particularly increased in diabetes and other age-related inflammatory or metabolic diseases, and in chronic kidney disease due to decreased excretion^1^. Accumulation of AGEs in tissues, including the brain, may lead to modification of proteins and of the extracellular matrix and to activation of inflammatory pathways by binding to the receptor for AGEs^3^. It is suggested that AGEs contribute to cognitive impairment^4^, dementia^5,6^, cerebral atrophy^7–9^, and to Alzheimer-related pathology^10–12^. For instance, previous studies showed higher concentrations of AGEs in the brain, cerebrospinal fluid, and serum of patients with Alzheimer’s disease (AD)^13–17^. Thus, AGE accumulation may have a role in the mechanisms linking diabetes to dementia^6,18^. Moreover, interactions of AGEs with *APOE* ε4, the most important genetic risk factor for dementia at older age, have been suggested^19,20^.

Tissue accumulation of AGEs can be estimated non-invasively as skin autofluorescence with an AGE Reader^21^, which may reflect AGE accumulation in tissues with low turnover, such as the brain. Skin autofluorescence measurement is based on fluorescent properties of AGEs and has been demonstrated to correlate with levels of both fluorescent and non-fluorescent AGEs in biopsy-derived skin tissue^21^. Levels of skin autofluorescence are increased in persons with diabetes and predict cardiovascular disease and mortality^22^. In addition, previous studies have shown that AGE accumulation in the skin is associated with worse cognition and with dementia cross-sectionally^19,23^. So far, no longitudinal studies have been conducted to study the association between AGE accumulation and the risk of dementia. Few studies investigated the association of AGE accumulation with brain volumetrics, as a preclinical marker of dementia, or with markers of cerebral small vessel disease^7–9^.

In this study, we determined the association of skin AGEs with the risk of dementia and whether they are related to measures of brain atrophy and of cerebral small vessel disease. In addition, we aimed to assess whether certain subgroups of participants, such as *APOE* ε4 carriers or persons with type 2 diabetes, might drive such associations.

## Methods and materials

### Study design

This study is embedded within the Rotterdam Study, a prospective population-based cohort designed to study the occurrence and determinants of diseases in the older population, as described previously^24^. Briefly, in 1990 all inhabitants aged 55 years or over from the district Ommoord in Rotterdam, the Netherlands, were invited to participate. The initial cohort comprised 7983 participants (subcohort RS-I) and was extended in 2000 with 3011 participants (subcohort RS-II) who had become 55 years of age or moved into the study district. In 2006, the cohort was further extended (subcohort RS-III) with 3932 participants aged 45 years or over. In total, the Rotterdam Study comprises 14,926 participants. Brain MRI scanning was performed in the Rotterdam Study population from 2005 onwards. The Rotterdam Study has been approved by the medical ethics committee according to the Population Study Act Rotterdam Study, executed by the Ministry of Health, Welfare and Sports of the Netherlands. All participants gave written informed consent. All methods were performed in accordance with the relevant guidelines and regulations.

### Study population

Skin autofluorescence was measured between 2013 and 2016 in 3009 participants from RS-I-6, RS-II-4, and RS-III-2. Participants with outlying skin autofluorescence levels (defined as mean ± 4 standard deviations (SDs); N = 8) were excluded. Of the remaining participants, 2929 were free of dementia (35 had dementia at the time of skin autofluorescence assessment, 44 had unknown dementia status) and were eligible for the current study. A subset of those participants also had a brain MRI scan which was acquired between 2013 and 2016 (N = 1504). For analyses with brain volumes, participants with cortical infarcts (N = 42) were excluded. Information on lacunes and microbleeds was available for 1476 participants. Hippocampus volume (sum of left and right) was available for 1104 participants.

### Measurement of skin AGEs

During the visit to the research center, skin autofluorescence was measured using the AGE Reader device (DiagnOptics B.V., Groningen, The Netherlands) based on the fluorescent property of AGEs. Briefly, approximately 4 cm^2^ of skin at the volar side of the dominant forearm was illuminated with an excitation light source from the AGE Reader with a peak wavelength of 370 nm. The device estimates skin AGEs based on the emission and reflection spectrum, which is converted through a software into numerical values reported in arbitrary units. Thus, an elevated skin autofluorescence score corresponds to a high tissue AGE level. This method has been validated against AGEs measured in skin biopsies from the same site where skin autofluorescence was measured^21^. Participants were asked not to use skin creams before the measurement. The mean of three consecutive measurements was used for analyses.

### Assessment of dementia

Participants were screened for dementia at baseline and subsequent center visits with the Mini-Mental State Examination and the Geriatric Mental Schedule organic level. Those with a Mini-Mental State Examination score < 26 or Geriatric Mental Schedule score > 0 underwent further investigation and informant interview, including the Cambridge Examination for Mental Disorders of the Elderly. In addition, the entire cohort was continuously under surveillance for dementia through electronic linkage of the study database with medical records from general practitioners and the regional institute for outpatient mental health care. Available information on clinical neuroimaging was used when required for diagnosis of dementia subtype. The final diagnosis was established by a consensus panel led by a consultant neurologist, according to standard criteria for dementia (using Diagnostic and Statistical Manual of Mental Disorders III-revised and Alzheimer’s Disease (AD) (using National Institute of Neurological and Communicative Disorders and Stroke-Alzheimer Disease and Related Disorders Association).

### Brain imaging

Brain MRI scanning was performed on a 1.5-Tesla MRI scanner (General Electric Healthcare, Milwaukee, USA) with an 8-channel head coil. Imaging acquisition included a high-resolution axial T1-weighted sequence, proton density-weighted sequence, a fluid attenuated inversion recovery sequence and a T2*-weighted gradient echo sequence were acquired. The scan protocol, sequence details and processing of MRI data in the Rotterdam study were previously described elsewhere^25^. Total intracranial and parenchymal volumes and volume of white matter hyperintensities were quantified via automated tissue segmentation. Quantification of brain volumetric measures was obtained by automated brain tissue segmentation based on a k-nearest neighbor algorithm. All segmentations were visually inspected and manually corrected when necessary. Total brain volume was defined as the sum of grey and white matter volume. Hippocampus volume was obtained by processing T1-weighted images with FreeSurfer (version 5.1)^26^. Visual evaluation of all scans was performed by trained raters to assess the presence of cortical infarcts, lacunes, and cerebral microbleeds.

For volumetric markers, we used total brain volume, grey matter volume, white matter volume, and hippocampal volume. Cerebral small vessel disease markers comprised white matter hyperintensity volume, presence of lacunes (yes/no), and presence of microbleeds (yes/no).

### Assessment of covariates

During home interviews, participants provided information on educational level, smoking status, alcohol use and medication use (antidiabetic medication, antihypertensive, and lipid lowering medication)^24^. Educational level was categorized as primary, lower, intermediate, or higher education. Smoking status was classified into never, current, or former. Alcohol use was categorized as no use or any use. At the research center, height and weight were measured and the body mass index (kg/m^2^) was computed. Blood pressure was measured in the sitting position on the right arm using a random-zero sphygmomanometer. Serum concentrations of glucose, total cholesterol, high-density lipoprotein cholesterol, triglycerides, and creatinine were measured in fasting blood samples during the previous center visit (2009–2013). Serum 25-hydroxyvitamin D levels were derived from earlier rounds (1997–2008) as it has not been measured afterwards. The estimated glomerular filtration rate (eGFR, mL/min/1.73 m^2^) was calculated using the Chronic Kidney Disease Epidemiology Collaboration equation^27^. Chronic kidney disease was defined as an eGFR less than 60 mL/min/1.73 m^2^. *APOE* was genotyped by polymerase chain reaction in RS-I and by biallelic TaqMan assay in RS-II and RS-III^28,29^. Participants were categorized as carries of no, one, or two ε4 alleles according to the *APOE* genotype. Type 2 diabetes was defined as fasting blood glucose > 7.0 mmol/L, use of antidiabetic medications, interview data, or as having type 2 diabetes according to general practitioners’ records.

### Statistical analyses

Baseline characteristics were described for the total study population, stratified into tertiles of skin autofluorescence, and for the participants with brain MRI available. Additionally, baseline characteristics are reported for age- and sex-balanced tertiles of skin autofluorescence, derived by regressing skin autofluorescence on age and sex and categorizing the residuals into tertiles. These balanced tertiles were solely used for the comparison of baseline characteristics and not for the further analyses. For descriptive purposes, we also created a scatterplot showing all individual measurements of skin autofluorescence by the age of participants. The associations of skin autofluorescence with the risk of dementia and AD were assessed using Cox proportional hazard models. In these models, skin autofluorescence was analyzed in two ways: per SD difference, and categorized into tertiles, with the lowest tertile as the reference. Follow-up started when skin autofluorescence was measured and ended at the date of dementia diagnosis, date of death, or end of the study period (January 1, 2020), whichever came first. We repeated the analyses after stratifying by *APOE* ε4 carrier status (carriers versus non-carriers), after stratifying by type 2 diabetes status, and after excluding participants with chronic kidney disease to investigate whether these subgroups drove the associations.

The associations between skin autofluorescence and brain imaging markers were determined using linear regression for continuous outcomes and logistic regression for dichotomous outcomes.

All analyses were adjusted for age, sex and subcohort (model 1). In model 2, we additionally adjusted for other potential confounders, namely fasting glucose levels, use of antidiabetic medication, educational level, *APOE* ε4 carrier status, smoking behavior, eGFR and 25-hydroxyvitamin D. In model 3, we also adjusted for potential confounders which—given the cross-sectional measurement of these variables with the measurement of skin autofluorescence—may actually be intermediates, namely systolic and diastolic blood pressure, total cholesterol level, high-density lipoprotein cholesterol level, triglyceride levels and the use of blood pressure and/or lipid-lowering medication. To evaluate whether age was sufficiently adjusted for, we tested whether additionally adjusting for age squared changed the results. Analyses with volumetric brain imaging measures were additionally adjusted for intracranial volume.

Missing data on covariates were imputed using fivefold multiple imputation (i.e., “multivariate imputation by chained equations” package in R statistical software version 3.6.3 [R Project for Statistical Computing]). Survival analyses were conducted using the “survival” package in R. Statistical testing was performed 2-sided with P < 0.05 considered significant.

## Results

Among the 2922 included participants the mean age was 72.6 years (SD 9.4) and 57% were women (Table 1). Skin autofluorescence levels were normally distributed with values ranging from 1.1 to 4.4 arbitrary units and a mean of 2.4 (SD 0.5). Levels increased with age of the participants (Supplementary Fig. 1), although this link was only seen for participants who did not develop dementia during follow-up. Participants in the highest tertile of skin autofluorescence were indeed older than participants in the lowest tertile (mean age 75.6 versus 69.9 years, P < 0.001, Table 1) and they were less often women (46% versus 67%). In addition, they were more often current smokers, had higher fasting glucose levels, higher prevalence of type 2 diabetes, and lower kidney function, even after regressing out the effects of age and sex (Supplementary Table 1). Participants with MRI (N = 1504) on average were slightly younger (mean age 71.2 [SD 9.2]) than the overall study population (Table 1).

**Table 1:** Baseline characteristics of the study population.

Characteristic	All participants				MRI subset
	Skin autofluorescence tertiles				
	Low	Medium	High	Total	
Sample size	1072	926	924	2922	1504
Age, years	69.9 (9.5)	72.8 (9.1)	75.6 (8.5)	72.6 (9.4)	71.2 (9.2)
Sex					
Men	351 (33)	415 (45)	502 (54)	1268 (43)	687 (46)
Women	721 (67)	511 (55)	422 (46)	1654 (57)	817 (54)
Rotterdam study subcohort					
RS-I	216 (20)	214 (23)	291 (31)	721 (25)	331 (22)
RS-II	311 (29)	354 (38)	384 (42)	1049 (36)	511 (34)
RS-III	545 (51)	358 (39)	249 (27)	1152 (39)	662 (44)
Educational level					
Primary	63 (6)	52 (6)	79 (9)	194 (7)	87 (6)
Lower	424 (40)	360 (40)	362 (40)	1146 (40)	556 (37)
Intermediate	314 (30)	276 (30)	290 (32)	880 (31)	465 (31)
Higher	259 (24)	222 (24)	181 (20)	662 (23)	376 (25)
Smoking					
Never	438 (41)	313 (34)	263 (29)	1014 (35)	525 (35)
Former	562 (53)	519 (56)	540 (59)	1621 (56)	828 (55)
Current	66 (6)	92 (10)	116 (13)	274 (9)	144 (10)
*APOE* ε4 alleles					
No allele	724 (72)	643 (73)	639 (74)	2006 (73)	1058 (74)
1 allele	256 (26)	212 (24)	203 (24)	671 (24)	335 (24)
2 alleles	22 (2)	22 (3)	18 (2)	62 (2)	28 (2)
Skin autofluorescence, arbitrary units	1.9 (0.2)	2.4 (0.1)	3.0 (0.3)	2.4 (0.5)	2.3 (0.5)
Systolic blood pressure, mmHg	139.0 (20.8)	140.9 (20.2)	140.4 (20.4)	140.0 (20.5)	138.8 (19.8)
Diastolic blood pressure, mmHg	78.0 (11.8)	76.9 (10.9)	74.1 (11.2)	76.4 (11.4)	77.0 (11.2)
Glucose, mmol/L	5.7 (0.9)	5.8 (1.1)	6.1 (1.6)	5.8 (1.2)	5.8 (1.1)
Total cholesterol, mmol/L	5.6 (1.1)	5.5 (1.1)	5.2 (1.1)	5.5 (1.1)	5.5 (1.1)
High-density lipoprotein cholesterol, mmol/L	1.6 (0.4)	1.5 (0.4)	1.4 (0.4)	1.5 (0.4)	1.5 (0.4)
Triglycerides, mmol/L	1.4 (0.8)	1.5 (0.7)	1.5 (0.7)	1.4 (0.7)	1.4 (0.8)
25-Hydroxyvitamin D, nmol/L	66.5 (27.5)	63.3 (28.1)	59.7 (28.3)	63.4 (28.1)	64.6 (28.2)
Estimated glomerular filtration rate, mL/min/1.73 m^2^	77.9 (13.1)	75.1 (14.0)	71.9 (15.1)	75.2 (14.2)	76.7 (13.6)
Chronic kidney disease	93 (9)	128 (14)	184 (21)	405 (14)	164 (11)
Diabetes	147 (14)	170 (18)	254 (28)	571 (20)	267 (18)
Medication					
Antidiabetic medication	52 (5)	80 (9)	148 (16)	280 (10)	109 (7)
Antihypertensive medication	494 (46)	476 (52)	568 (62)	1538 (53)	725 (49)
Lipid lowering medication	358 (34)	356 (39)	422 (46)	1136 (39)	561 (38)
Brain MRI tissue volume, mL					
Total brain volume					915.6 (98.5)
White matter volume					390.0 (58.7)
Grey matter volume					525.7 (55.5)
Hippocampus volume					6.6 (0.8)
Markers of cerebral small vessel disease					
White matter hyperintensity volume, mL					8.3 (11.1)
Lacunar infarcts					100 (7)
Microbleeds					369 (25)

Numbers are means (standard deviation) or numbers (percentages).

Missing values (% in total study population) per variable: education (1.4%), smoking (0.4%), *APOE* ε4 genotype (6.3%), systolic blood pressure (0.3%), diastolic blood pressure (0.3%), glucose (3.9%), total cholesterol (3.9%), high-density lipoprotein cholesterol (3.9%), 25-hydroxyvitamin D (7.9%), estimated glomerular filtration rate (4.3%), chronic kidney disease (4.3%), diabetes (0.2%), medication (0.8%).

In total, 123 participants developed dementia during a median of 4.3 years of follow-up (interquartile range 3.3–5.3), of whom 98 had AD. Higher levels of skin autofluorescence per SD associated with an increased risk of dementia (hazard ratio (HR) 1.21 [95% confidence interval (CI) 1.01–1.46) and of AD (HR 1.19 [0.97–1.47]), adjusted for potential confounders (model 2). Participants in the highest skin autofluorescence tertile had a 1.4-fold higher risk of dementia and a 1.3-fold higher risk of AD, compared to participants in the lowest tertile (model 2 adjusted HRs 1.42 [95% CI 0.88–2.29] and 1.29 [0.76–2.19], respectively). Additional adjustment for other cardiovascular risk factors (model 3) did not change these results. Table 2 shows the associations per SD and by tertile of skin autofluorescence with the risk of dementia and of AD using the different models for adjustment.

**Table 2:** Associations of skin autofluorescence with the risk of dementia.

Skin autofluorescence	n/N	Hazard ratio (95% confidence interval)		
		Model 1	Model 2	Model 3
	All-cause dementia			
Per standard deviation	123/2922	1.24 (1.05–1.47)	1.21 (1.01–1.46)	1.23 (1.02–1.48)
Tertiles				
Low	30/1072	Reference	Reference	Reference
Medium	32/926	0.93 (0.56–1.55)	0.90 (0.54–1.51)	0.92 (0.55–1.54)
High	61/924	1.53 (0.97–2.42)	1.39 (0.86–2.23)	1.42 (0.88–2.29)
	Alzheimer’s disease			
Per standard deviation	98/2922	1.21 (0.99–1.47)	1.19 (0.97–1.47)	1.20 (0.97–1.48)
Tertiles				
Low	25/1072	Reference	Reference	Reference
Medium	26/926	0.89 (0.50–1.56)	0.88 (0.50–1.55)	0.90 (0.51–1.59)
High	47/924	1.37 (0.82–2.29)	1.27 (0.75–2.15)	1.29 (0.76–2.19)

Hazard ratios resulting from analyses per standard deviation difference and by tertile of skin autofluorescence. The hazard ratios can be interpreted as the relative risk of dementia associated with a standard deviation higher value of skin autofluorescence or, for the per tertile results, the relative risk for participants with a medium or high value compared to a low value, while all covariates remain the same. Model 1 is adjusted for age, sex and subcohort. Model 2 additionally adjusts for potential confounders (education, smoking, *APOE* ε4 status, estimated glomerular filtration rate, 25-hydroxyvitamin D, glucose and use of antidiabetic medication). Model 3 additionally adjusts for potential confounders/mediators (systolic blood pressure, diastolic blood pressure, total cholesterol, high-density lipoprotein cholesterol, triglycerides, use of antihypertensive and/or lipid lowering medication).

**Figure 1:**
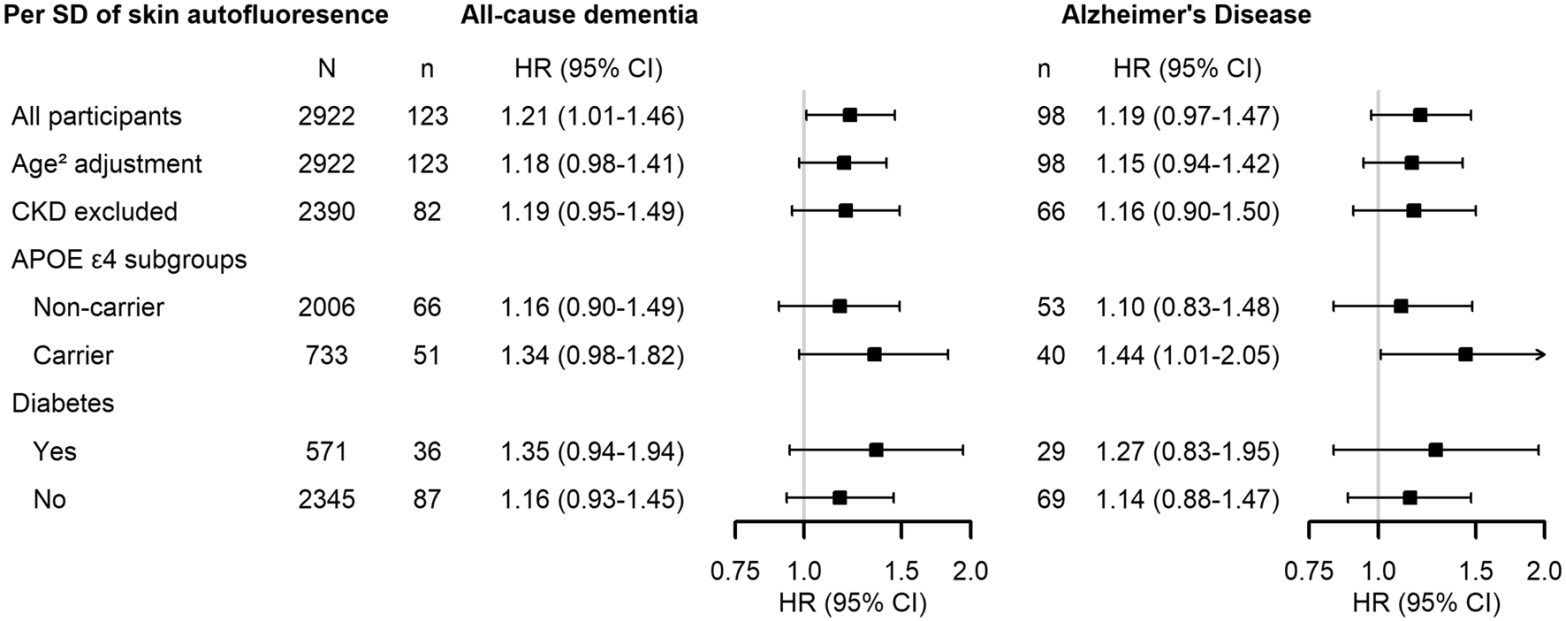
Skin autofluorescence in association with the risk of dementia and Alzheimer’s disease in subgroups of the study population. *CKD* chronic kidney disease, *CI* confidence interval, *HR* hazard ratio, *SD* standard deviation. Hazard ratios per standard deviation difference of skin autofluorescence. All hazard ratios are adjusted for age, sex, subcohort, education, smoking, *APOE* ε4 status (except for the *APOE* ε4 stratified analyses), estimated glomerular filtration rate, 25-hydroxyvitamin D, glucose and use of antidiabetic medication.

The increased risks were somewhat more pronounced in *APOE* ε4 carriers (HR per SD higher 1.34 [0.98–1.82] for all-cause dementia; 1.44 [1.01–2.05] for AD, Fig. 1 and Supplementary Table 2) and in persons with type 2 diabetes (HR 1.35 [0.94–1.94] and 1.27 [0.83–1.95]). Note, though, that formal interaction terms were not significant for these stratifications. Exclusion of participants with CKD did not substantially change the effect sizes, nor did further adjustment for age squared.

**Figure 2:**
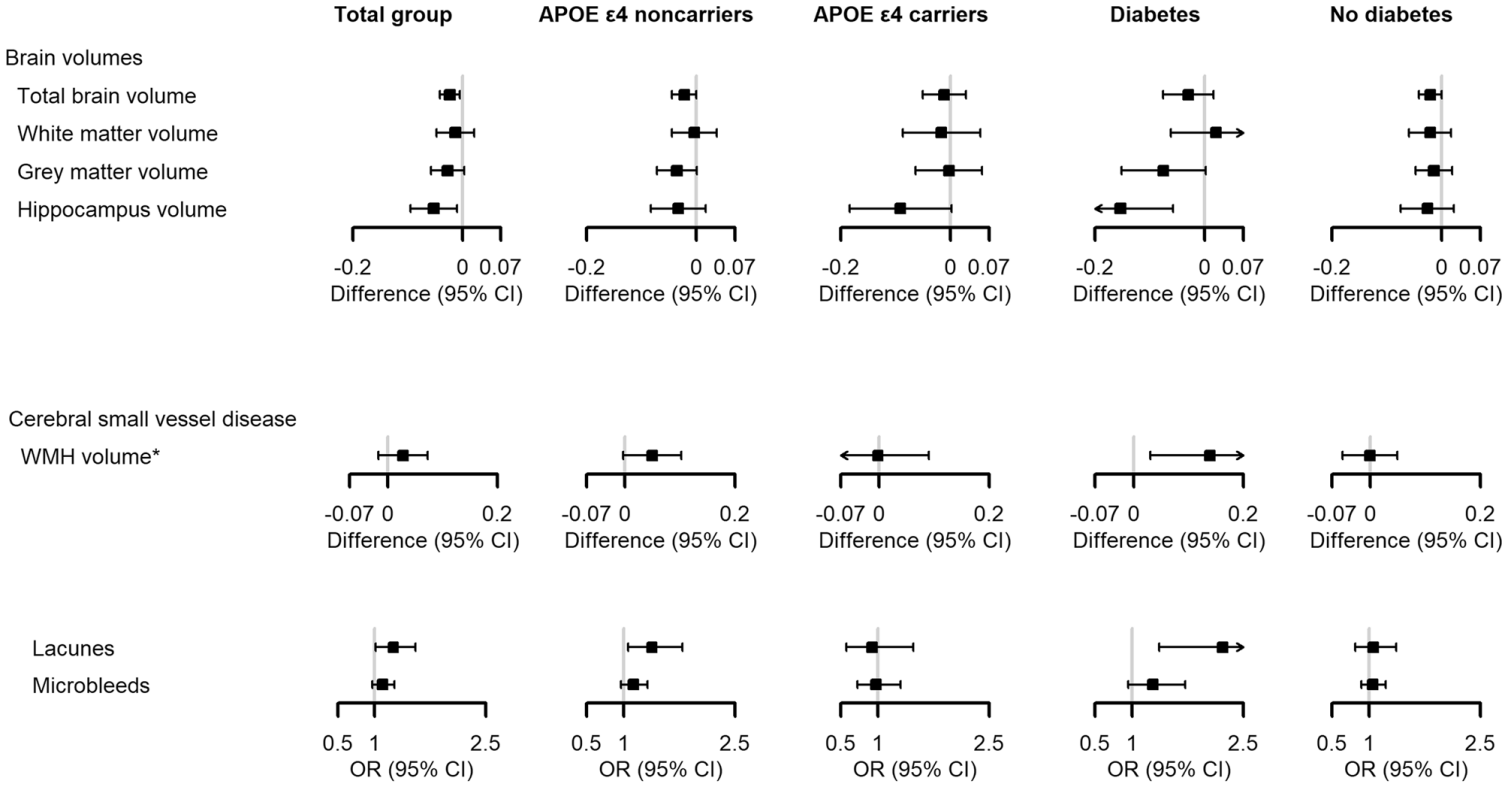
Skin autofluorescence in association with volumetric brain MRI measures and measures of small vessel disease. *CI* confidence interval, *OR* odds ratio, *SD* standard deviation, *WMH* white matter hyperintensity. Estimates are z-scores for differences in volumetric measures or odds ratios per standard deviation differences of skin autofluorescence. All estimates are adjusted for age, sex, subcohort, intracranial volume, education, smoking, *APOE* ε4 status (except for the *APOE* ε4 stratified analyses), estimated glomerular filtration rate, 25-hydroxyvitamin D, glucose and use of antidiabetic medication. *White matter hyperintensity volumes are logarithmically transformed.

Participants with higher skin autofluorescence also had smaller total brain volumes (adjusted difference in z-score per SD − 0.02 [− 0.04; 0.00]), non-significantly smaller grey matter volumes (− 0.03 [− 0.06; 0.00]) and smaller hippocampus volumes (− 0.05 [− 0.10; − 0.01]), but not white matter volumes (− 0.01 [− 0.05; 0.02], Fig. 2, details in Supplementary Tables 3 and 4).

In addition, they tended to have higher white matter hyperintensity volumes (0.03 [− 0.02; 0.07]) and to more often have microbleeds and lacunes (odds ratios: 1.11 [0.97–1.27] and 1.25 [1.01–1.55]).

Associations of skin autofluorescence with brain MRI measures were mainly present in participants with type 2 diabetes and in *APOE* ε4 non-carriers, except for hippocampus volume, which was associated with skin autofluorescence in both *APOE* ε4 carriers and non-carriers, although not statistically significantly. Again, the results did not change after excluding participants with CKD or with additional adjustment for age squared (Supplementary Tables 3 and 4).

## Discussion

We found that higher skin autofluorescence, reflecting long-term accumulation of AGEs, is associated with a higher risk of all-cause dementia and of AD, independently of age and several other potential confounders. These associations were more pronounced in *APOE* ε4 carriers and in participants with type 2 diabetes. Skin AGE levels were also associated with a smaller total brain volume, grey matter volume and hippocampus volume, and with presence of lacunes, and non-significantly with white matter hyperintensity volume and with presence of microbleeds.

Our results are in line with existing cross-sectional literature that found higher levels of AGEs in the skin and in the brain, plasma, serum or urine of persons with dementia or cognitive impairment^4,8,16,19,23^. The results of our study add to the current literature by linking AGEs to dementia in a longitudinal setting, thereby supporting the hypothesis that AGEs could contribute to the etiology of dementia.

Involvement of AGEs in dementia pathology was first described in 1994 and was based on co-localization of AGEs with senile plaques and neurofibrillary tangles in the brains of patients with AD^30–32^. More recent literature suggests that AGEs in the brain induce inflammation and oxidative stress, resulting in synaptic dysfunction and neuronal damage, and contribute to deposition and accumulation of dementia related pathologies both intracellularly (e.g. tau) and extracellularly (e.g. amyloid β)^5,6,10,11,14,33,34^. In that way, AGEs might underlie the increased risk of dementia among persons with diabetes^18^. These effects may result from direct toxic effects of AGEs and from interaction of AGEs, or other ligands, including amyloid β, with the receptor for AGEs (RAGE), subsequently triggering inflammatory pathways and, in turn, upregulation of RAGE^3^. Interestingly, RAGE also has a role in the transport of amyloid β into the brain across the blood–brain barrier^11,35,36^. Inhibition of RAGE has been proposed to decrease pathogenic events in AD. RAGE antagonists reduced amyloid β levels and improved learning and memory deficits in mouse models^37–40^, but, so far, results from a trial in patients with mild to moderate AD have been inconclusive^41,42^.

The finding that AGE levels associate with measures of brain atrophy, and particularly with lower grey matter volumes, is in agreement with previous smaller studies as well^8,9^, although associations with decreased hippocampus volumes and increased cerebral small vessel disease were not previously reported. Such brain changes might thus partially mediate an effect of AGEs on dementia, especially in persons with diabetes. However, further investigation is needed to assess the causality of these findings.

*APOE* ε4 genotype is the strongest genetic risk factor for AD, with the mechanisms likely related to its role in lipid metabolism^43^. An interaction between AGEs and apoE has been proposed, given colocalization in the brain and binding activity of apoE to AGE-modified proteins^20^. In this study, *APOE* ε4 genotype modified the associations of skin AGEs and dementia such that the associations were more pronounced among carriers. Contrastingly, AGE levels among carriers were not associated with most brain atrophy measures or with measures of cerebral small vessel disease. Other biological pathways linking AGEs and dementia in *APOE* ε4 carriers are, therefore, more plausible. For example, apoE4 has higher affinity for AGEs than apoE3 and this apoE-AGE interaction could contribute to plaque formation in AD^20^. Similarly, a synergistic effect of *APOE* ε4 and diabetes on the risk of AD was previously found, which, according to the authors, may be mediated by AGEs^44^.

Diabetes is an important risk factor for both AGE accumulation and dementia^45,46^. Our results suggest that AGEs relate to (preclinical) dementia, particularly among persons with type 2 diabetes and to a lesser extent among persons without type 2 diabetes. The latter may be partially explained by their lower absolute AGE levels^16^, or the presence of other compensative mechanisms, such as vessel health, that makes persons without diabetes less susceptible to the effects of AGE accumulation.

Strengths of our study include the large population in whom AGE levels, relevant other variables such as *APOE* ε4 and diabetes, and subsequent dementia incidence were assessed. AGE accumulation was measured in the skin, which is thought to reflect accumulation in other long-lived tissues^47^, such as the brain^48^, Some limitations also need to be discussed. First, due to the median follow-up of 4.3 years and in view of the long preclinical phase of dementia, inferences about the direction of the effects should be made with caution. Second, we only measured AGEs with fluorescent properties. Yet, these measurements were shown to correlate with levels of non-fluorescent AGEs as well and are thus considered a marker of the total skin AGE pool^21^. Third, our results were restricted to an elderly population of European ancestry and generalizability may thus be limited.

In conclusion, our findings suggest a role of AGE accumulation in the pathophysiology of dementia, which might contribute to the link between diabetes and dementia. Further research is warranted to determine whether reducing AGE accumulation, and relatedly, RAGE expression and activation, could be protective against dementia. Finally, future studies may explore whether *APOE* ε4 carriers are more susceptible to AGE related pathology and how *APOE* ε4 and AGEs might have a joint effect.

### Supplementary Information

Supplementary Information.

## Data Availability

Data can be obtained upon request. Requests should be directed towards the management team of the Rotterdam Study (secretariat.epi@erasmusmc.nl), which has a protocol for approving data requests. Because of restrictions based on privacy regulations and informed consent of the participants, data cannot be made freely available in a public repository.
