# Supplementary material for: Skin autofluorescence, reflecting accumulation of advanced glycation end products, and the risk of dementia in a population-based cohort
Source: Sci Rep. 2024 Jan 13;14:1256. doi: 10.1038/s41598-024-51703-6 (PMC10787742; doi:10.1038/s41598-024-51703-6)
Supplement: Supplementary file 1 — Supplementary Information. [file 41598_2024_51703_MOESM1_ESM.pdf]

Supplemental material

Skin autofluorescence, reflecting accumulation of advanced glycation end products, and the risk of dementia in a population-based cohort

Sanne S. Mooldijk, BSc[1], Tianqi Lu, MSc[1,2], Komal Waqas, MD[2], Jinluan Chen, PhD[1,2], Meike W. Vernooij, MD, PhD[1,3], M. Kamran Ikram, MD, PhD[1,4], M. Carola Zillikens, MD, PhD[2], M. Arfan Ikram, MD, PhD[1]*

[1] Department of Epidemiology, Erasmus University Medical Center Rotterdam, Rotterdam, The Netherlands

[2] Department of Internal Medicine, Erasmus University Medical Center Rotterdam, Rotterdam, The Netherlands

[3] Department of Radiology and Nuclear Medicine, Erasmus MC University Medical Center, Rotterdam, the Netherlands

[4] Department of Neurology, Erasmus University Medical Center Rotterdam, Rotterdam, The Netherlands

*Address correspondence to: Prof. M. Arfan Ikram, Department of Epidemiology, Erasmus University Medical Center, PO Box 2040, 3000 CA, Rotterdam, The Netherlands. E-mail: m.a.ikram@erasmusmc.nl.

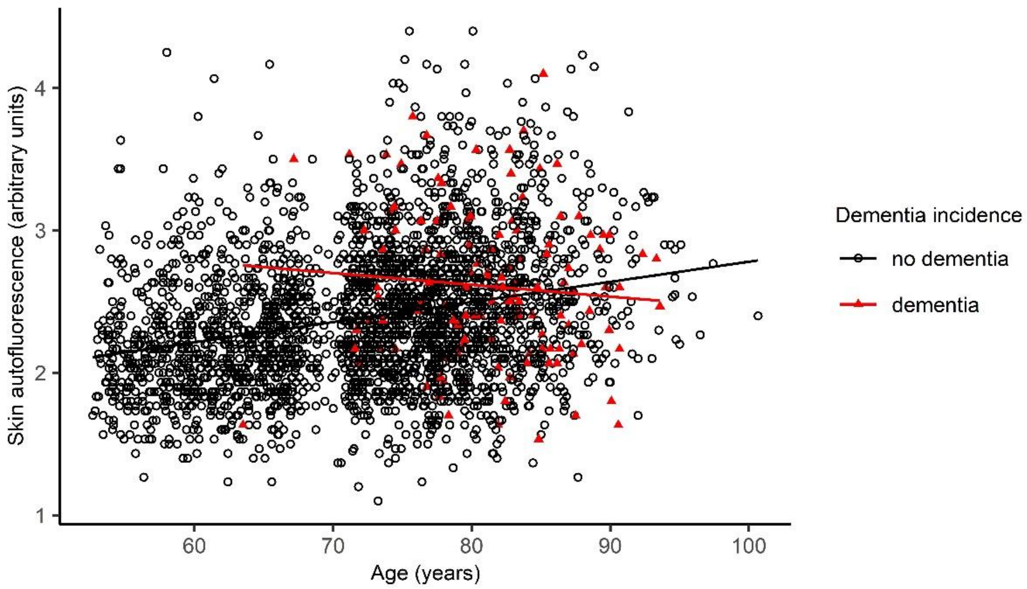

Supplementary figure 1. Skin autofluorescence by age and dementia incidence

Skin autofluorescence levels of individual participants at baseline. The lines were computed using linear regressions (skin autofluorescence ~ age). Among participants without dementia during follow-up (black line and circles [N=2799]), the skin autofluorescence levels increased with age (0.014 arbitrary units per year [95% confidence interval 0.012 to 0.016]); among participants who were diagnosed with dementia during follow-up (red line and triangles [N=123]), they did not (-0.008 [-0.025 to 0.009]).

Supplementary Table 1. Baseline characteristics of the study population by age- and sex-balanced tertiles of skin autofluorescence

Characteristic	Age- and sex-balanced tertiles		
	**Low**	**Medium**	**High**
Sample size	974	974	974
Age, years	73.1 (9.5)	71.5 (9.4)	73.2 (9.1)
Sex			
Men	430 (44)	418 (43)	420 (43)
Women	544 (56)	556 (57)	554 (57)
Rotterdam Study subcohort			
RS-I	278 (29)	210 (22)	233 (24)
RS-II	341 (35)	332 (34)	376 (39)
RS-III	355 (36)	432 (44)	365 (37)
Educational level			
Primary	57 (6)	49 (5)	88 (9)
Lower	367 (38)	381 (40)	398 (41)
Intermediate	300 (31)	292 (30)	288 (30)
Higher	236 (25)	240 (25)	186 (19)
Smoking			
Never	380 (39)	334 (34)	300 (31)
Former	540 (56)	548 (56)	533 (55)
Current	50 (5)	88 (9)	136 (14)
APOE ε4 alleles			
No allele	670 (73)	667 (73)	669 (74)
1 allele	227 (25)	228 (25)	216 (24)
2 alleles	22 (2)	21 (2)	19 (2)
Skin autofluorescence, arbitrary units	1.9 (0.2)	2.3 (0.2)	2.9 (0.4)
Systolic blood pressure, mm Hg	140.9 (20.6)	139.7 (20.5)	139.4 (20.4)
Diastolic blood pressure, mm Hg	77.0 (11.7)	77.3 (11.2)	75.0 (11.3)
Glucose, mmol/L	5.7 (0.9)	5.8 (1.1)	6.0 (1.6)
Total cholesterol, mmol/L	5.5 (1.1)	5.5 (1.1)	5.3 (1.1)
High-density lipoprotein cholesterol, mmol/L	1.5 (0.4)	1.5 (0.4)	1.5 (0.4)
Triglycerides, mmol/L	1.4 (0.7)	1.4 (0.8)	1.5 (0.7)
25-hydroxyvitamin D, nmol/L	67.8 (27.9)	63.7 (27.3)	58.3 (28.3)
Estimated glomerular filtration rate, mL/min/1.73 m^2	75.6 (13.2)	75.9 (13.8)	74.0 (15.6)
Chronic kidney disease	118 (12)	113 (12)	174 (19)
Diabetes	156 (16)	166 (17)	249 (26)
Medication			
Antidiabetic medication	55 (6)	72 (7)	153 (16)
Antihypertensive medication	496 (51)	477 (49)	565 (59)
Lipid lowering medication	358 (37)	371 (38)	407 (42)

Numbers of means (standard deviation) or numbers (percentages). Age- and sex-balanced tertiles were derived by regressing skin autofluorescence on age and sex and categorizing the residuals into tertiles.

Supplementary Table 2. Skin autofluorescence in association with the risk of dementia and Alzheimer's Disease in subgroups of the study population

Per SD skin autofluorescence	n/N	Hazard ratio (95% confidence interval)			
		**Unadjusted**	**Model 1**	**Model 2**	**Model 3**
		**All-cause dementia**			
Total sample	123/2922	1.56 (1.33-1.83)	1.24 (1.05-1.47)	1.21 (1.01-1.46)	1.23 (1.02-1.48)
Additional adjustment for age[2]	123/2922	-	1.19 (1.01-1.42)	1.18 (0.98-1.41)	1.19 (0.99-1.43)
Persons without CKD	82/2390	1.60 (1.31-1.94)	1.23 (1.00-1.53)[a]	1.19 (0.95-1.49)	1.23 (0.97-1.54)
APOE ε4 non-carriers	66/2006	1.57 (1.27-1.95)	1.27 (1.01-1.61)	1.16 (0.90-1.49)	1.14 (0.88-1.47)
APOE ε4 carriers	51/733	1.66 (1.29-2.14)	1.27 (0.95-1.70)	1.34 (0.98-1.82)	1.39 (1.02-1.90)
Persons with diabetes	36/571	1.59 (1.16-2.18)	1.32 (0.94-1.86)	1.35 (0.94-1.94)	1.39 (0.94-2.03)
Persons without diabetes	87/2345	1.51 (1.25-1.83)	1.20 (0.98-1.48)	1.16 (0.93-1.45)	1.17 (0.94-1.47)
		**Alzheimer's Disease**			
Total sample	98/2922	1.53 (1.28-1.83)	1.21 (0.99-1.47)	1.19 (0.97-1.47)	1.20 (0.97-1.48)
Additional adjustment for age[2]	98/2922	-	1.16 (0.95-1.41)	1.15 (0.94-1.42)	1.17 (0.95-1.44)
Persons without CKD	66/2390	1.54 (1.23-1.92)	1.19 (0.93-1.52)	1.16 (0.90-1.50)	1.20 (0.92-1.56)
APOE ε4 non-carriers	53/2006	1.51 (1.18-1.93)	1.19 (0.90-1.57)	1.10 (0.83-1.48)	1.10 (0.82-1.47)
APOE ε4 carriers	40/733	1.67 (1.25-2.22)	1.29 (0.93-1.79)	1.44 (1.01-2.05)	1.45 (1.02-2.07)
Persons with diabetes	29/571	1.50 (1.05-2.14)	1.25 (0.85-1.86)	1.27 (0.83-1.95)	1.31 (0.80-2.12)
Persons without diabetes	69/2345	1.50 (1.21-1.86)	1.18 (0.93-1.49)	1.14 (0.88-1.47)	1.15 (0.89-1.49)

CKD, chronic kidney disease; SD, standard deviation.
Hazard ratios per standard deviation difference of skin autofluorescence. Tertiles are age-balanced. Model 1 is adjusted for age, sex and subcohort. Model 2 additionally adjusts for potential confounders (education, smoking, *APOE* ε4 status, estimated glomerular filtration rate, 25-hydroxyvitamin D, glucose and use of antidiabetic medication). Model 3 additionally adjusts for potential confounders/mediators (systolic blood pressure, diastolic blood pressure, total cholesterol, high-density lipoprotein cholesterol, triglycerides, use of antihypertensive and/or lipid lowering medication). [a] $p \geq 0.05$.

Supplementary Table 3. Skin autofluorescence and volumetric MRI brain measures

**Skin autofluorescence, per standard deviation**	**Total brain volume**	**White matter volume**	**Grey matter volume**	**Hippocampus volume**
**Total group**	Difference in z-scores (95% confidence interval)			
Unadjusted	-0.09 (-0.14; -0.04)	-0.11 (-0.16; -0.06)	-0.04 (-0.09; 0.01)	-0.17 (-0.23; -0.11)
Model 1	-0.03 (-0.05; -0.01)	-0.02 (-0.06; 0.01)	-0.03 (-0.06; 0.00)[b]	-0.06 (-0.10; -0.03)
Model 2	-0.02 (-0.04; 0.00)[a]	-0.01 (-0.05; 0.02)	-0.03 (-0.06; 0.00)[b]	-0.05 (-0.10; -0.01)
Model 3	-0.02 (-0.04; 0.00)[a]	-0.01 (-0.04; 0.02)	-0.03 (-0.06; 0.00)[b]	-0.05 (-0.10; -0.01)
**Subgroup/sensitivity analyses**				
Additional adjustment for age^2	-0.03 (-0.05; -0.01)	-0.01 (-0.05; 0.02)	-0.03 (-0.06; 0.00)[b]	-0.05 (0.10; -0.01)
Persons without CKD	-0.02 (-0.04; 0.00)[a]	-0.02 (-0.06; 0.02)	-0.02 (-0.06; 0.01)	-0.06 (-0.11; -0.01)
APOE ε4 non-carriers	-0.02 (-0.04; 0.00)[a]	0.00 (-0.05; 0.04)	-0.04 (-0.07; 0.00)[b]	-0.03 (-0.08; 0.02)
APOE ε4 carriers	-0.01 (-0.05; 0.03)	-0.02 (-0.09; 0.06)	0.00 (-0.06; 0.06)	-0.09 (-0.18; 0.01)
Persons with diabetes	-0.03 (-0.08; 0.02)	0.02 (-0.06; 0.10)	-0.08 (-0.15; 0.00)[b]	-0.15 (-0.25; -0.06)
Persons without diabetes	-0.02 (-0.04; 0.00)[b]	-0.02 (-0.06; 0.02)	-0.01 (-0.05; 0.02)	-0.03 (-0.07; 0.02)

CI, confidence interval; CKD, chronic kidney disease.
Estimates are z-score differences of brain volumes per standard deviation differences of skin autofluorescence. Model 1 is adjusted for age, sex, subcohort and intracranial volume. Model 2 additionally adjusts for potential confounders (education, smoking, *APOE* ε4 status, estimated glomerular filtration rate, 25-hydroxyvitamin D, glucose and use of antidiabetic medication). Model 3 additionally adjusts for potential confounders/mediators (systolic blood pressure, diastolic blood pressure, total cholesterol, high-density lipoprotein cholesterol, triglycerides, use of antihypertensive and/or lipid lowering medication). Subgroup analyses are adjusted using model 2. [a] $p<0.05$. [b] $p\geq0.05$.

Supplementary Table 4. Skin autofluorescence and measures of cerebral small vessel disease

**Skin autofluorescence, per standard deviation**	**WMH volume (log transformed)**	**Microbleeds**	**Lacunes**
**Total group**	β (95% CI)	OR (95% CI)	OR (95% CI)
Unadjusted	0.20 (0.15; 0.25)	1.23 (1.10; 1.38)	1.51 (1.25; 1.81)
Model 1	0.05 (0.01; 0.09)	1.09 (0.96; 1.24)	1.26 (1.03; 1.55)
Model 2	0.03 (-0.02; 0.07)	1.11 (0.97; 1.27)	1.25 (1.01; 1.55)
Model 3	0.04 (-0.01; 0.08)	1.10 (0.96; 1.26)	1.27 (1.02; 1.58)
**Subgroup/sensitivity analyses**			
Additional adjustment for age^2	0.03 (-0.02; 0.07)	1.11 (0.97; 1.27)	1.25 (1.01; 1.55)
Persons without CKD	0.02 (-0.03; 0.07)	1.16 (1.00; 1.35)[b]	1.22 (0.94; 1.59)
APOE ε4 non-carriers	0.05 (0.00; 0.10)[b]	1.13 (0.96; 1.32)	1.38 (1.06; 1.80)
APOE ε4 carriers	0.00 (-0.10; 0.09)	0.92 (0.57; 1.48)	0.97 (0.72; 1.30)
Persons with diabetes	0.14 (0.03; 0.25)	1.28 (0.95; 1.72)	2.22 (1.36; 3.61)
Persons without diabetes	0.00 (-0.05; 0.05)	1.05 (0.90; 1.22)	1.05 (0.81; 1.37)

CI, confidence interval; CKD, chronic kidney disease; WMH, white matter hyperintensity; OR, odds ratio. Estimates are z-score differences of logarithmic transformed white matter lesion volumes (β) or odds ratios per standard deviation differences of skin autofluorescence. Model 1 is adjusted for age, sex, subcohort and intracranial volume. Model 2 additionally adjusts for potential confounders (education, smoking, *APOE* ε4 status, estimated glomerular filtration rate, 25-hydroxyvitamin D, glucose and use of antidiabetic medication). Model 3 additionally adjusts for potential confounders/mediators (systolic blood pressure, diastolic blood pressure, total cholesterol, high-density lipoprotein cholesterol, triglycerides, use of antihypertensive and/or lipid lowering medication). Subgroup analyses are adjusted using model 2. [a] $p<0.05$. [b] $p\geq0.05$.
